# Upper-Limb Prosthetic Myocontrol: Two Recommendations

**DOI:** 10.3389/fnins.2015.00496

**Published:** 2016-01-06

**Authors:** Claudio Castellini, Raoul M. Bongers, Markus Nowak, Corry K. van der Sluis

**Affiliations:** ^1^German Aerospace Center (DLR), Institute of Robotics and MechatronicsWessling, Germany; ^2^Center for Human Movement Sciences, University of Groningen, University Medical Center GroningenGroningen, Netherlands; ^3^Department of Rehabilitation Medicine, University of Groningen, University Medical Center GroningenGroningen, Netherlands

**Keywords:** upper-limb prosthetics, machine learning, myocontrol, functional assessment

## Background

The loss of the upper limb (hand, elbow, shoulder) is one of the most disabling conditions a person can ever experience, leading to a severe loss of daily-life functionality. In Assistive Robotics, the problem of designing, constructing and testing a *prosthetic* robotic arm/hand is formidable: it must enforce high dexterity—many degrees of freedom, fast motions, large workspace and high precision—while respecting strict requirements on weight, power consumption, noise and appearance. Nowadays, relatively dexterous elbow, wrist and hand prostheses are commercially available that get more and more sold and implanted; *prosthetic control* is then the problem of enabling the amputee to control these artifacts to their best extent (Farina et al., 2014): finely enough to master their dexterity and naturally enough to let the patient feel that the prosthesis is a new part of her body. This refers to the notion of *embodiment* as discovered, for instance, in the “rubber hand illusion” (Botvinick and Cohen, 1998).

Such a control system must perform the desired actions quickly, precisely, safely and reliably, at the same time providing sensory feedback to the subject (Bongers et al., 2012). Now, despite decades of academic research, it is amazing how far from this ideal situation we still are, even neglecting sensorial feedback: detecting the patient's intent and transforming it into effective control signals is still a largely open problem. Most research effort today is focused on advancing the process of interpreting the signals generated by the remnant muscle activations in the stump, be it via surface electromyography (Merletti et al., 2011) or more visionary techniques (Castellini et al., 2014); this interpretation, given the unpredictability of signals recorded from human subjects and the potentially endless variety of the situations in which a prosthesis is supposed to work, is enforced using machine learning. Nevertheless, machine-learning-based myocontrol has not yet made the final step to the clinics, to the prosthetic market, and in general it is not in use in the daily life of the standard amputee. Why is it so? As of today, *the control system is the bottleneck* (Jiang et al., 2012), the main failure being unreliability—the ever-impending possibility that the system will take the wrong decision, potentially leading to catastrophic results.

Unreliability could be tackled in many ways; for instance, more sensors together with data fusion would enable a finer intent detection; miniaturization and full wearability would enable a smoother usage of the prosthesis. Following our previous research in the field, we hereby make two recommendations: firstly, we suggest to use incremental learning, leading to the interaction of the patient with the control system, rather than batch calibration; secondly, we sketch future assessment protocols targeted at measuring the benefits of this very interaction, in order to encourage researchers to test their prototypes on end-users.

## Interaction instead of calibration

Most machine learning methods build an approximant function (*model*) given a set of example input–output values. This is the case in upper-limb prosthetics too: the model being sought for maps signals such as the ones described above to control signals for the prosthesis. Traditionally (Fougner et al., 2012) this is enforced via batch calibration: a somewhat large set of examples (training set) is collected at the beginning of a session; a model is generated; and it is then used to predict the desired prosthesis configuration, picking it from a predetermined, finite and usually small set of possibilities (classification). Typically, such a system predicts “hand opening,” “elbow flexing” and so on; by batch calibration we refer to this necessity of gathering a large training set, and to the fact that the calibration of the system happens once at the beginning and can hardly be modified in due course.

Some of the limitations inherent to classification have been tackled by introducing *simultaneous and proportional control* (Radhakrishnan et al., 2008; Jiang et al., 2009), whereby the model predicts the activation level of each degree of freedom of the prosthesis rather than one of a finite number of predefined configurations. This breakthrough does not, however, solve the rigidity inherent in batch calibration: the training set must contain all possible sources of change in the input signals in advance. This is essentially unfeasible (Castellini, 2015): typical such conditions are sweating, relative skin/muscle motion, change in the musculoskeletal configuration due to body movement, arm stretching, etc., and, last but not least, the potential necessity of learning new configurations as time goes by—that is, conditions which are an essential part of the life of an amputee. For example, consider the (very different) upper-limb position and orientation while grasping an object from a shelf above one's head, as opposed to grasping something on the ground.

To solve this problem, either we build a model of the arm/hand/body system and of the environment to interact with, such that the training set will fairly represent the input space; or we gather more data *on demand*, whenever the system fails or the subject wants to teach the prosthesis a new pattern. If the machine learning method we use allows for *incremental learning*, one can, and in our opinion should, follow this latter route. We refer to incremental learning as to the possibility to modify the model at any moment during the usage without requiring a reevaluation of the previous model, but only the incorporation of novel information into it; updating the model, e.g., for different postural variations, should be performed on demand. Updating needs be easy and fast, allowing for an intuitive, natural and reliable interaction with the prosthesis; an example of such a method has been shown in Gijsberts et al. (2014).

Incremental learning should also allow for downgrading/removing data that has become obsolete. Human subjects do indeed get better and better at controlling their own signals along time (Powell and Thakor, 2013); in this case, after a while, the old data won't reflect any longer the current state of control and would have a negative influence on the overall control performance.

## A novel functional assessment protocol

The proposed shift of paradigm in myoelectric control calls for a novel way to measure the outcomes of its deployment in the target population, to foster discussion about its applicability and comparison with existing systems. Furthermore, translation and implementation of the device in the upper-limb amputees population needs such results. Two main questions arise: what to measure, i.e., on which domains do we expect change or improvement, and what the purpose of the measurement is de Vet et al. (2011); moreover, do we intend to measure capacity (what a patient can do) or performance (what a patient does do?) (Buffart et al., 2006) *Capacity* is mostly measured in a laboratory setting, whereas *performance* is a reflection of acting in a home or work environment.

To come to an accepted evaluation tool, the psychometric properties of the measurement must be taken into account: is the measurement instrument *valid, reliable*, and *responsive?* (Terwee et al., 2007) The level at which measurements take place is also important: the *conceptual model of health* (World Health Organization, 2001) should be used to ensure that measurements are performed at all functional levels (Stallinga, 2015). In this model the functioning of the upper-limb amputee is examined by looking at components of body function (signals, pain, embodiment), activities (what the patient does in daily life) and participation (what relationships the patient is involved in). Functioning is influenced by environmental as well as personal factors.

*At the level of body functions* it is desirable to measure the signals used in the human-machine interface, the body kinematics to record compensatory movements, physical and cognitive fatigue, embodiment, and smoothness/fluency/velocity of the upper limb movements. *At the level of activities*, uni- and bi-manual execution of daily tasks should be evaluated; the tasks should be increasingly relevant to the patient as the developmental stages of the system progress along with the ability of the patient to use the system itself. The tasks should have a gradual increase in difficulty, which can be assigned to the task itself but also to the context in which the task is to be executed. Relevant existing measurement instruments, which have previously been designed for the target population, can be used to compile relevant tasks, such as SHAP (Light et al., 2002), UBET (Bagley et al., 2006), UNB (Sanderson and Scott, 1985), ACMC (Hermansson et al., 2005), or in case of questionnaires, UEFS-OPUS (Burger et al., 2008), or even more general questionnaires, such as DASH (Hudak et al., 1996). Lastly, *at the level of participation*, questionnaires can be used to evaluate work participation and work productivity with the new device or participation in leisure activities. Concepts derived from health-related quality-of-life evaluations should also be considered: is the patient's quality of life improving when using the new device? The widely used Short Form-36 (SF-36) (Tarlov et al., 1989) could be considered for this issue or the IPA (Impact on Participation and Autonomy), which reflects participation and autonomy in different domains (Cardol et al., 1999). Finally, the patient and/or the researcher should rate satisfaction with the different levels of device development and the effects of the device on different levels of functioning. Visual analog scales or Likert scales could be considered to cover this issue.

In general, such a measurement instrument should be both researcher- and patient-based—both the researcher and the patient should assess the performance of the control system; and in our case, in which the patient/system interaction is to be evaluated, scoring at the upper end of the instrument (ceiling effect) is particularly undesired. The tasks selected for the assessment should be assigned a *difficulty factor*, to be determined in collaboration with the target population. Besides this, the relevancy of the tasks or questions needs attention: if an upper-limb amputee is asked to perform activities or answer questions regarding prosthesis use and she never uses her prosthesis during daily life, the validity of the measurement instrument is at stake. Thus, in the developmental stage of a measurement instrument, involvement of the target population and pilot testing are a prerequisite. We also advise to measure *capacity* during the try-out phase in the laboratory, and *performance* when the system has reached its (pre)final stage, when patients should be observed during their personal daily life.

## Discussion

*Incremental learning leads to interaction with the prosthesis*, which in the first place can be used to correct apparent failures of the control system. By “interaction” here we mean a structured approach of the interplay of the patient with the prosthesis—the feeling that she is teaching the robot what she wants. This will, in our opinion, lead to an increased feeling of symbiosis and embodiment, even though the sensorial feedback is still technologically unripe. Lastly, we believe it is essential to exploit the phenomenon of *reciprocal learning*—the possibility that not only the prosthesis adapts to the patient's signals, which is inherent in the usage of machine learning, but as well that she learns to use it better and better along time. This entails that her signals change and, from the point of view of the control system, improve, i.e., they get more repeatable and separated in the input space.

In view of this, we also need a novel instrument capable of measuring, both numerically and subjectively, the fruitfulness of the patient/prosthesis interaction. The patient's increasing proficiency in performing daily-life activities using the prosthesis must be quantifiable in a valid way. To this aim we propose decisions that have to be made when developing a measuring instrument, such as what will be measured with which purpose, as well as whether the focus is on capacity or performance. Moreover, a measuring instrument has to be developed that is valid, reliable and responsive.

## Author contributions

All authors contributed equally to the writing of this article and share the same opinions expressed in it.

### Conflict of interest statement

The authors declare that the research was conducted in the absence of any commercial or financial relationships that could be construed as a potential conflict of interest.
